# Oxidative Stress in Alcohol Abuse: An Unfortunately Still Open Question

**DOI:** 10.3390/antiox13080934

**Published:** 2024-08-01

**Authors:** Marco Fiore

**Affiliations:** Institute of Biochemistry and Cell Biology, IBBC-CNR, c/o Campus Internazionale, Via E. Ramarini, 32, 00015 Rome, Italy; marco.fiore@cnr.it

As the guest editor of this Special Issue “Alcohol-Induced Oxidative Stress in Health and Disease” of *Antioxidants* (https://www.mdpi.com/journal/antioxidants/special_issues/alcohol accessed on 29 July 2024), I am pleased to introduce this collection of papers exploring the multidimensional and complex relationship between alcohol consumption and oxidative stress, alongside the consequent health effects. Alcohol, a substance embedded in human culture for millennia, causes both instants of conviviality and severe health risks. This dichotomy makes alcohol an undeniable topic of interest for scientific studies.

Alcohol consumption is prevalent, and while moderate consumption is often socially tolerable and sometimes even associated with certain health benefits, the negative effects of alcohol abuse are intense. These outcomes range from instantaneous impacts such as neurocognitive damage, nausea, and hangovers, to long-term effects including dependence, liver and brain injury, and an elevated risk of various tumors. Fundamental to many of these detrimental consequences is alcohol-induced oxidative stress, an issue that this Special Issue aims to disclose.

Oxidative stress results from a disproportion between the production of reactive oxygen species (ROS) and reactive nitrogen species (RNS), and the body’s capability to cleanse these reactive intermediates or restore the resulting injury. Ethanol metabolism plays a crucial role in this imbalance. Indeed, alcohol metabolism mostly occurs in the liver through enzymatic paths involving alcohol dehydrogenase (ADH), cytochrome P450 2E1 (CYP2E1), and catalase. These routes produce ROS as by-products, which lead to oxidative stress if not effectively neutralized by the endogenous antioxidant systems.

The function of mitochondria and the microsomal ethanol-oxidizing system (MEOS) in this activity is central. Mitochondrial dysfunction caused by chronic alcohol abuse intensifies ROS production, leading to cellular loss. Furthermore, alcohol-induced activation of CYP2E1 potentiates ROS and RNS production, further moving the redox balance towards a pro-oxidant level. This oxidative stress negatively disrupts several organs, particularly the brain and liver, leading to disorders such as neurodegeneration and alcoholic liver disease (ALD).

This Special Issue collects novel research and review papers that expand our knowledge of the processes by which alcohol-induced oxidative stress participates in health and disease. The contributions focus on both clinical and preclinical outcomes, offering a fascinating view of current understanding and forthcoming research directions (Contributions 1–5).

One of the recurrent topics in this Special Issue is the sophisticated relationship between alcohol-induced oxidative stress and the body’s antioxidant defense systems. Some papers discuss how chronic alcohol abuse harms the functionality of the main antioxidant enzymes such as catalase, glutathione peroxidase (GPx), and superoxide dismutase (SOD). These changes potentiate oxidative impairment, eliciting the progression of disorders such as alcoholic cardiomyopathy and ALD (Contributions 2–3).

Another topic of this Issue regards the toxic effect of alcohol on the fetus if consumed during pregnancy. Likewise, alterations in oxidative stress induced by alcohol may also severely affect the newborn through epigenetic mechanisms (Contributions 6–7).

Another important topic to focus on is the possible therapeutic role of antioxidants in counteracting alcohol-induced oxidative injury. Studies included in this Issue explore various antioxidant compounds, including synthetic antioxidants and natural antioxidants like polyphenols (Contributions 8–9). These reports offer promising evidence that increasing the body’s antioxidant ability can reduce some of the harmful effects of alcohol abuse. However, the intricacy of alcohol-induced oxidative stress requires a more direct approach to antioxidant treatment, considering aspects such as timing, dosage, and the definite paths involved (Contributions 10–11).

While noteworthy progress has been made in the comprehension of alcohol-induced oxidative stress, several questions persist. Future research should be conducted to unravel the detailed molecular mechanisms by which alcohol affects redox homeostasis. There is a crucial necessity for more comprehensive studies on the relationship between several ROS and RNS species and their specific cellular targets (Contributions 1, 3).

Additionally, translating preclinical findings into efficient clinical therapies remains a challenge. More accurate clinical trials are needed to assess the efficacy and safety of antioxidant treatments in humans. It should be noted that personalized medicine methods, considering individual differences in antioxidant capacity and alcohol metabolism, could also improve the efficacy of these interventions.

In conclusion, this Special Issue of *Antioxidants* underlines the subtle role of oxidative stress in alcohol-related health topics and focuses on potential possibilities for treatments. The combined efforts of investigators contributing to this Issue may expand our knowledge and provide novel expectations for mitigating alcohol’s negative effects through targeted antioxidant approaches.

I extend my sincere gratitude to all the contributors, reviewers, and the editorial team for their priceless roles in the foundation of this Special Issue. I do believe that the insights disclosed from these studies will stimulate further studies to improve clinical practices intended to decrease the burden of alcohol-induced disorders.

